# Federated, Accessible, Inclusive, and Responsive Virtual Biorepository: Delphi Consensus Study

**DOI:** 10.2196/87458

**Published:** 2026-11-12

**Authors:** Amy Price, Layla Abdulbaki, Julia Poje, Judith Giri, Zoe Steinberg, Thomas Jaenisch, May Chu

**Affiliations:** 1 Dartmouth Health, Lebanon, NH, United States; 2 Center for Global Health, Colorado School of Public Health, University of Colorado, 13001 East 17th Place, Mail Stop B119, Aurora, CO, 80045, United States, 1 303 724 4869; 3 Oakland University, Rochester, MI, United States; 4 Infectious and Tropical Medicine, Heidelberg Institute for Global Health, Heidlberg University, Heidelberg, Germany

**Keywords:** public health, infectious disease, biorepository, virtual biorepository, pandemic sample sharing, epidemiology, pandemic preparedness, equity

## Abstract

**Background:**

The reemergence of the Zika virus, the novel coronavirus, and new influenza strains highlighted the urgent need for an organized forum to access specimens to advance research, diagnostics, and vaccine development. During the COVID-19 pandemic, gaps in supply chains, communication, and standards between local (federated) and centralized systems cost time and lives. The FAIR-VBS (Federated, Accessible, Inclusive, and Responsive Virtual Biorepository) was proposed to connect communities with samples, address specimen-sharing challenges, and promote inclusion and equity.

**Objective:**

This study aims to enable equitable contribution, sharing, and access to specimens while addressing the complexities involved. The objective is to build stakeholder consensus on prioritized actions, tools, and responsibilities to establish a functional and practical FAIR-VBS.

**Methods:**

A Delphi process was conducted online to gather stakeholder input and develop a prioritized action plan for the FAIR-VBS. Respondents were recruited using snowball sampling, and questions were informed by participatory workshops, feedback, outreach efforts, and discussions to identify gaps and best practices. The Delphi process consisted of 2 rounds: an initial ranking informed by stakeholder input and a second round to refine priorities based on the panel feedback. Items were evaluated using a standardized 5-point Likert scale and retained if prioritized by at least 70% of respondents rating an item 4 or higher and no more than 15% rating an item below 2, with descriptive statistics applied. Advisory board discussions supplemented the virtual process to capture nuances, while qualitative data were analyzed using participatory action research methods.

**Results:**

The Delphi process evaluated 34 cumulative items across 2 rounds to identify critical gaps and priorities in biorepository systems. In the first round, 22 out of 30 initial items successfully reached consensus. Panelists organically proposed 4 additional items during the first round; upon evaluation in the second round, 2 of these newly proposed items reached consensus, addressing benefit-sharing challenges (11/15, 73%) and the prioritization of whole blood, plasma, and serum samples (40/42, 95%). Culturally, peripheral blood mononuclear cells were supported as a secondary priority (13/18, 72%). In total, 24 items achieved consensus across both rounds, while 10 items overall did not. Respondents overwhelmingly supported the hybrid FAIR-VBS, which combines federated and centralized systems, offering local convenience with virtual oversight (34/41, 83%). Cost recovery for services was supported by all respondents (43/43, 100%), with a preference for a sliding fee scale to subsidize capacity building in low- and middle-income countries (40/42, 95%).

**Conclusions:**

Respondents strongly supported the FAIR-VBS to enable equitable access to specimens, services, and benefits. They emphasized the need for clarity on benefit-sharing, regulatory, and ethical frameworks to ensure effective implementation and global inclusivity. A fair and equivalent system to enhance participation and operational continuity is being discussed.

## Introduction

Access to high-quality, well-characterized specimens that reflect global diversity, including populations from low- and middle-income countries, is critical for advancing research, diagnostics, and vaccine development [1]. Despite the evident need during outbreaks such as Zika (2016) and COVID-19 (2019), and concerns around evolving pathogen threats, a sustainable and trusted global resource for these samples has yet to be realized. While the concept is not new, progress has been hindered by challenges in regulatory and ethical implementation, as well as concerns over tangible benefits and long-term sustainability, which have discouraged partners from advancing these efforts [1]. Disparities in diagnostic kit performance across high- and low-income settings underscore the importance of globally representative specimens [2,3]. The COVID-19 pandemic, in particular, exposed delays in accessing essential samples and hampered timely research and development [4].

Historically, international collections for infectious diseases have been centralized within large, well-funded institutions and biorepositories, often excluding local contributors and geographically diverse populations [5]. The pandemic amplified inequities in diversity, equity, and inclusion within these systems [2,3]. To address these challenges, we propose a virtual biorepository that connects local repository hubs for efficient, equitable, and accessible sample sharing [1].

The FAIR-VBS (Federated, Accessible, Inclusive, and Responsive Virtual Biorepository) is designed to be agile and inclusive, combining the strengths of grassroots initiatives with sensitivity to local contexts as “an additional repository type” to the standard centralized systems. By reducing the need for a centralized multinational facility, the FAIR-VBS reduces overhead but could be limited by the types and uses of its specimens, so we needed to find consensus on how to advance equity while defining the types of accessible samples with simple storage needs that would allow a global partner with limited resources to join the effort. Importantly, our work will complement other major international biobanking initiatives such as the WHO BioHub, H3Africa, and the Global Biobank Alliance; multiple individuals from these groups and others are mentioned in our *Acknowledgments*.

This study uses an international online Delphi process to achieve consensus and solve challenges collaboratively [6,7]. The Delphi method offers several advantages: it (1) empowers underresourced populations by fostering inclusion and reimagining global biorepository systems, (2) amplifies local voices to define the benefits and sustainability of the FAIR-VBS, (3) creates a roadmap for addressing end-user priorities, (4) reduces power imbalances through anonymous contributions, and (5) accommodates cultural and geographic diversity among respondents.

The need for a systematic, inclusive approach to specimen access is urgent, particularly as global health threats like Zika, emerging coronaviruses, and influenza variants resurface [5,8]. Biorepositories are essential for providing qualified, annotated samples that advance diagnostics, disease prediction, and prevention efforts [1,4].

Our aim is to develop actionable recommendations for building and maintaining a virtual biorepository while fostering ongoing consensus as the initiative evolves. This grassroots effort aims to facilitate scientific exchange, equitable specimen sharing, and global collaboration as a public good. By aligning with successful international partnerships, the FAIR-VBS seeks to be an inclusive, additive, and sustainable model for a multinational community. Using the Delphi method, we plan to identify practices that promote biorepository availability, good governance, and long-term sustainability [9]. Our protocol and supplementary materials are available on the Open Science Framework [10] and in Multimedia Appendix 1.

## Methods

### Aims and Objectives

We aimed to identify prioritized actions, tools, and responsibilities necessary to establish a functional and FAIR-VBS. Specifically, our objectives were to develop actionable, consensus-based recommendations for building and maintaining an inclusive, diverse, and equity-driven FAIR-VBS and to generate ongoing findings as the project evolves to ensure continued alignment with stakeholder needs and global priorities.

### Preparation Work

The research team collected information from professional contacts and organizations; conducted surveys and interviews [1]; held workshops; searched the literature; set up websites; and connected with others in the field, including biorepository experts, public health practitioners, policymakers, industry leaders, funders, students, and members of the public. The research team conducted internal audits to systematically evaluate the effectiveness and alignment of its activities with the project’s goals. These audits reviewed the outcomes of workshops and stakeholder engagements, assessed gaps in the literature and expert input, and identified actionable next steps [1]. Through this reflective process, we deliberated and concluded that an online Delphi process could offer a practical approach to bridge cultural, linguistic, and geographical divides; foster autonomous local consensus [11]; and ensure cost-effectiveness [12]. Figure 1 shows the timeline for FAIR-VBS and the steps we used to prepare for the Delphi process.

**Figure 1.**
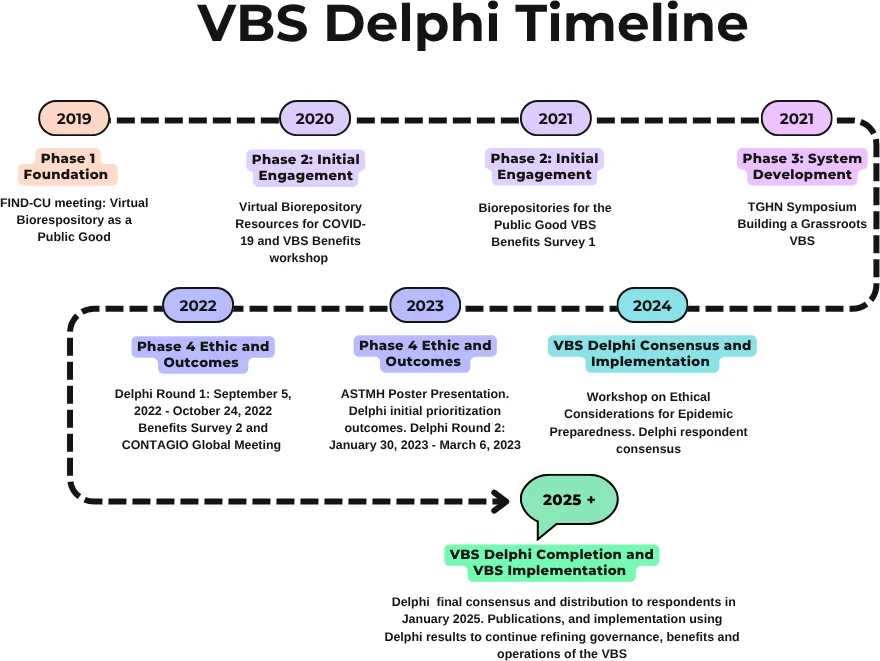
Preparatory FAIR-VBS (Federated, Accessible, Inclusive, and Responsive Virtual Biorepository) Delphi timeline by phases. ASTMH: American Society of Tropical Medicine and Hygiene; CONTAGIO: Cohorts Network To Be Activated Globally In Outbreaks; CU: University of Colorado; FIND: Foundation for Innovative New Diagnostics; TGHN: The Global Health Network; VBS: virtual biorepository system.

### The Delphi Process

The Delphi method is a structured, iterative communication tool designed to facilitate consensus-building, forecasting, or shared decision-making. Through a series of rounds, whether delivered onsite or by using virtual survey software, respondents evaluate and prioritize presented questions, typically participating in at least 2 rounds [9]. The Delphi method was offered online through a secure interface. After each round, participants received an anonymous summary of the group’s input, enabling them to refine and prioritize their previous responses based on others’ feedback. This iterative process narrows the range of variation until common ground is reached. The underlying principle is that structured group input yields more accurate and actionable results than unstructured discussions [6].

### Study Design

#### The Mixed Methods Qualitative Bridge

While a traditional Delphi process is fundamentally a quantitative mechanism designed to measure consensus on predetermined metrics, it lacks an inherent qualitative capacity to explore the underlying reasons behind item failure or to capture organic, participant-driven innovations. To bridge this structural limitation, this study used a sequential mixed methods participatory action research design that embedded a formal qualitative content analysis engine between the quantitative survey rounds.

Open-ended comment text fields were appended to every quantitative item. When an item failed to meet the strict quantitative consensus threshold in round 1, the 5-member coding team systematically analyzed the qualitative text fields using a unified coding framework to uncover hidden semantic ambiguities, structural anxieties, and cross-cultural friction points. This qualitative bridge allowed the steering group to actively coproduce and rephrase items based directly on stakeholder insights. Additionally, this bridge provided the mechanism to capture and code the 4 entirely new items proposed organically by the panel in round 1, moving them into the quantitative voting pipeline for round 2. By embedding this qualitative bridge, the study evolved from a passive tracking survey into an agile, iterative, and truly participant-driven consensus process. All participant comments are included in Multimedia Appendix 1.

#### FAIR-VBS Online Delphi Process

The FAIR-VBS initiative was informed by global webinars, workshops, and feedback sessions involving participants from Africa, Latin America, Southeast Asia, and Europe. Contributors included biobank experts, blood bank consultants, public health specialists, researchers, and diagnostics manufacturers.

To shape grassroots, equitable approaches for specimen sharing, we used a mixed methods, sequential participatory action research design [13] centered on a multiple round online Delphi. This process was built on insights from workshops, ongoing sample collection efforts, and stakeholder discussions to frame the Delphi questions. We piloted the questions based on key needs identified from feedback from stakeholders and consultative workshops [1], which highlighted three target areas:

1. Types of specimens to share: Identifying priority specimens for global research and diagnostics
2. Solutions to barriers: Addressing logistical, regulatory, and ethical practices and cultural challenges in specimen sharing
3. Benefits and sustainability: Articulating the value and long-term viability of equitable specimen-sharing practices

The Delphi process engaged all stakeholders to rank, refine, and reach a consensus on these critical issues. Figure 2 provides a visual representation of the Delphi methodology and process.

**Figure 2.**
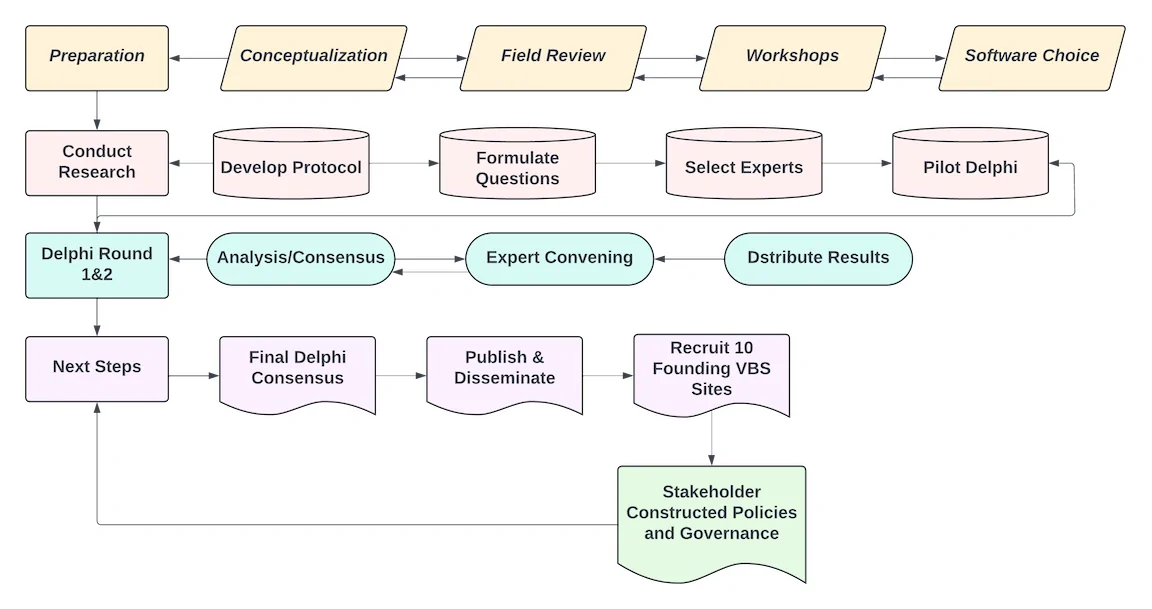
Virtual biorepository online Delphi process.

### Advisory Group and Steering Group

The Delphi development was guided by an advisory group specializing in infectious diseases, diagnostic manufacturing, and research methodology. The advisory group provided strategic oversight for the study design and its implementation. The advisory group and the researchers, together, formed the steering group to make decisions for the process. To ensure clarity, the Delphi questions were also reviewed by graduate students and advocates.

### Recruitment

Fifty-seven respondents were recruited using snowball sampling to ensure a diverse and inclusive panel. Stakeholders included public health experts, researchers, clinicians, community members, policymakers, regulators, and industry representatives, as well as respondents from 6 continents.

### Online Delphi Rounds

The Delphi process comprised 2 rounds, with each round serving a distinct purpose in the development and evaluation of the items:

1. Round 1 (exploratory phase): Respondents scored and commented on 30 initial items and were invited to propose additional questions. Items (n=40) were added by respondents, then reviewed and coded by 2 research team members to ensure relevance.
2. Round 2 (evaluative phase): Respondents reviewed results, including advisory group comments from round 1, and voted again on unresolved items. Respondents scored the 4 additional items proposed during round 1.

A consensus was reached after 2 rounds, and the results were reviewed and accepted by the advisory group. All results, including respondent comments with identifiers removed, were compiled and shared with participants. Consensus was defined as at least 70% of responses rating an item 4 or higher and no more than 15% rating an item below 2 on a 1 to 5 scale.

### Delphi Response Preparation

The responses were scored and prioritized using a Likert scale, with a 5-point format [14]. Respondents rated their level of agreement with each statement, with higher scores reflecting stronger agreement. This approach enabled a nuanced understanding of consensus among experts participating in the Delphi process. To further enrich the findings, qualitative content analysis was conducted. Five researchers (AP, LA, MC, JG, and JP) independently reviewed and categorized the textual responses provided by respondents, identifying themes, key concepts, and recurring opinions [6,12]. They reached a consensus on these interpretations, and the deidentified comments and analysis were presented to the steering group and then to the respondents. This contextual guidance informed the development of round 2 [15].

This method proved valuable in assessing how consensus evolves by considering levels of agreement or disagreement expressed in the comments alongside quantitative data, such as mean ratings or consensus thresholds. The analysis and all deidentified feedback were shared with respondents after the first and second rounds, fostering transparency and enabling a reflective and shared understanding of the group’s perspectives. All respondents and the steering group were presented with round 2 results. Following the Delphi rounds, a consensus conference was held via videoconferencing with the advisory group to finalize recommendations and define key next steps.

### Ethical Considerations

Ethical approval was obtained from the University of Oxford Central University Research Ethics Committee (CUREC; application reference number R58550/RE001 [16]). Informed consent was secured from all participants through a secure digital informed consent process. Contributors to the research, at their discretion, could be named as coauthors in the publication while maintaining the confidentiality of their data. To maximize transparency while maintaining participant privacy, all quantitative metrics and descriptive textual feedback were completely deidentified and anonymized prior to steering group appraisal and round-to-round dissemination. No participant compensation or financial incentives were provided for completion of the Delphi survey panels. Authors disclosed competing interests in accordance with ICMJE (International Committee of Medical Journal Editors) guidelines [17]. All data were securely stored online with restricted access limited to project-dedicated personnel.

### Data Collection and Research Reporting

Invitation letters, recruiting materials, forms, and Delphi questions were piloted by volunteers internally and externally. The CHERRIES (Checklist for Reporting Results of Internet E-Surveys [18]) guideline was used to structure and report the survey aspects of the study. The ACCORD (Accurate Consensus Reporting Document) was used to guide our methods for reporting the Delphi process [15].

### Patient and Public Involvement

The respondents and the authorship team spanned multiple continents and brought diverse perspectives to the study. This included partners and stakeholders with an interest in the FAIR-VBS, some of whom had personal experiences during the COVID-19 pandemic. Additionally, the study engaged research, public health, and medical students, as well as members of the public, all of whom actively contributed to the study design, protocol development, Delphi question testing, data analysis, and manuscript coauthorship.

### Dissemination

The FAIR-VBS research will be shared with repository groups; policymaking agencies; regional, national, and international conferences; government agency briefing sessions; the WHO BioHub; social media sites; and on the institutional websites of coauthors and members of our advisory group. Results will be open access and publicly available with a DOI. They will be shared with all respondents ahead of publication.

## Results

### Delphi Consensus Results

The Delphi process evaluated a cumulative total of 34 unique items across 2 operational rounds to identify critical gaps and priorities in biorepository systems. Over the entire life cycle of the study, a final system consensus was achieved for 24 out of 34 items, while 10 items overall did not meet the consensus criteria.

In round 1, the questionnaire evaluated 30 initial items, with 22 successfully meeting the consensus threshold. During this exploratory first round, panel participants organically proposed 4 additional items. These 4 new items were refined and presented to the cohort in round 2, where 2 achieved full consensus. These late-consensus items explicitly addressed institutional benefit-sharing challenges (11/15, 73% agreement) and the prioritization of whole blood, plasma, and serum samples (40/42, 95% agreement; Tables 1 and 2; Figure 3).

**Table 1.** The FAIR-VBS (Federated, Accessible, Inclusive, and Responsive Virtual Biorepository) Delphi results, round 1.

Main theme	Question	Answers or additional descriptions	Number of respondents in agreement above threshold^a^/total number of respondents replied to the question (% in agreement)
A. Questions assessing general interest	Overall, how interested are you in access to specimens?	Not applicable	42/47 (89%)
	Overall, how interested are you in biorepository operations?	Not applicable	40/47 (85%)
	Overall, do you think a grassroots effort is supportive of other concurrent efforts?	Not applicable	38/47 (81%)
	Rank support for combined federal and centralized systems	Combination of centralized and federated	37/47 (78%)
B. Questions about specimens	What types of specimens should be prioritized for accrual, storage, and sharing?	Whole blood, plasma, serum^b^	42/44 (95%)
	How should samples be prioritized for accrual, storage, and sharing?	Longitudinal: serial samples from confirmed cases	40/44 (91%)
		Prevaccination and 2 weeks postvaccination	37/44 (84%)
		Regionally with known endemic disease	36/44 (82%)
	How important is the availability of high volumes (>10 ml) of specimens for the following uses?	Important for reference or calibration use	34/34 (100%)
	Should there be minimal quality specimen standards and criteria for inclusion in the FAIR-VBS?	Yes	41/44 (93%)
	Which data elements are the highest priority for inclusion as a minimum to accompany specimens?	Specimen description, source and type, primary, or derivative, etc	41/41 (100%)
		Collection and handling conditions	41/45 (91%)
		Storage temperature and freeze-thaw cycles	38/45 (84%)
	Would you agree to a ranked system of specimens based on use, specimen quality, and data?	Yes	41/42 (98%)
	Which of the following criteria are most important to determining the tier of a specimen?	Accessibility (can be shipped from country)	36/42 (86%)
		Completeness of clinical data	33/42 (79%)
		Tests performed to characterize specimen	32/42 (76%)
	Which of the following criteria are most important to determining the tier of a specimen?	Handling and storage conditions	32/42 (76%)
C. Questions about common barriers	If you maintain specimens, does the ability to ship samples impact your ability to share?	Yes	39/43 (91%)
	Would you be able to share specimens designated for immune-based diagnostics or vaccine development?	Yes	35/38 (92%)
	Would you be willing to participate in defining policies for access and use of specimens?	Yes	33/39 (84%)
	Interest in FAIR-VBS generic templates for material transfer agreement (MTA) and material transfer data agreements (MTDAs)	Yes	36/40 (90%)
	Which of the following are important to include in such generic templates?	Ensure specimens are used for approved or designated purpose	40/42 (95%)
		Ensure specimens are transferred to other collaborators (third parties) by approval	38/42 (90%)
		Acknowledging the owners of specimens in publications and presentations	35/42 (83%)
		As a provider, you reserve the decision to share or not with the requesting party	30/42 (71%)
		Provision for tracking specimens (chain of custody) and their use	30/42 (71%)
D. Questions about benefits	No consensus, move to second Delphi round for additional information	Ask for clarification in round 2^b^	Not applicable
E. Questions about services	Would you support a cost recovery plan for services?	Yes	43/43 (100%)
	The following are some of the services we are considering. Rank each from highly desirable to least desirable	Reference panels (for calibration, proficiency, etc)	34/43 (79%)
		Protocols and templates (collection, storage, best practices, etc)	33/43 (77%)
		Infrastructure logistics (shipping, cold chain, equipment, etc)	33/43 (77%)
	Would you recommend academic, commercial, LMIC^c^, or other sliding fee scales for services?	Yes	40/42 (95%)
	Please rank again, of the following models for biorepositories, central, federated, or combined (repeat from general interest)	Combination of centralized and federated	34/41 (83%)

a Threshold for analysis: items were included if prioritization reached more than 70% agreement and discarded if fewer than 15% of respondents provided a response.

b Move to round 2 for further decision and consensus.

c LMIC: low- and middle-income country.

**Table 2.** The FAIR-VBS (Federated, Accessible, Inclusive, and Responsive Virtual Biorepository) Delphi results, round 2.

Main theme (from Table 1)	The 2 questions that did not receive consensus in the first round now posed to the second round for clarification	Answers to the questions or additional descriptions	Number of respondents in agreement above threshold^a^/total number of respondents replied to the question (% in agreement)
B. Specimens	Aside from whole blood, plasma and serum as first choice, what is the next ranked specimen for FAIR-VBS to collect?	Peripheral mononuclear blood cells	13/18 (72%)
D. Benefits	What is the single most important benefit to you?	Capacity building (39%) with networking and SOPs^b^ (28% each)	None reached threshold
	Are there challenges to receiving benefits you selected?	Yes	11/15 (73%)

a Threshold for analysis: items were included if prioritization reached more than 70% agreement and discarded if fewer than 15% of respondents provided a response.

b SOPs: standard operating procedures.

**Figure 3.**
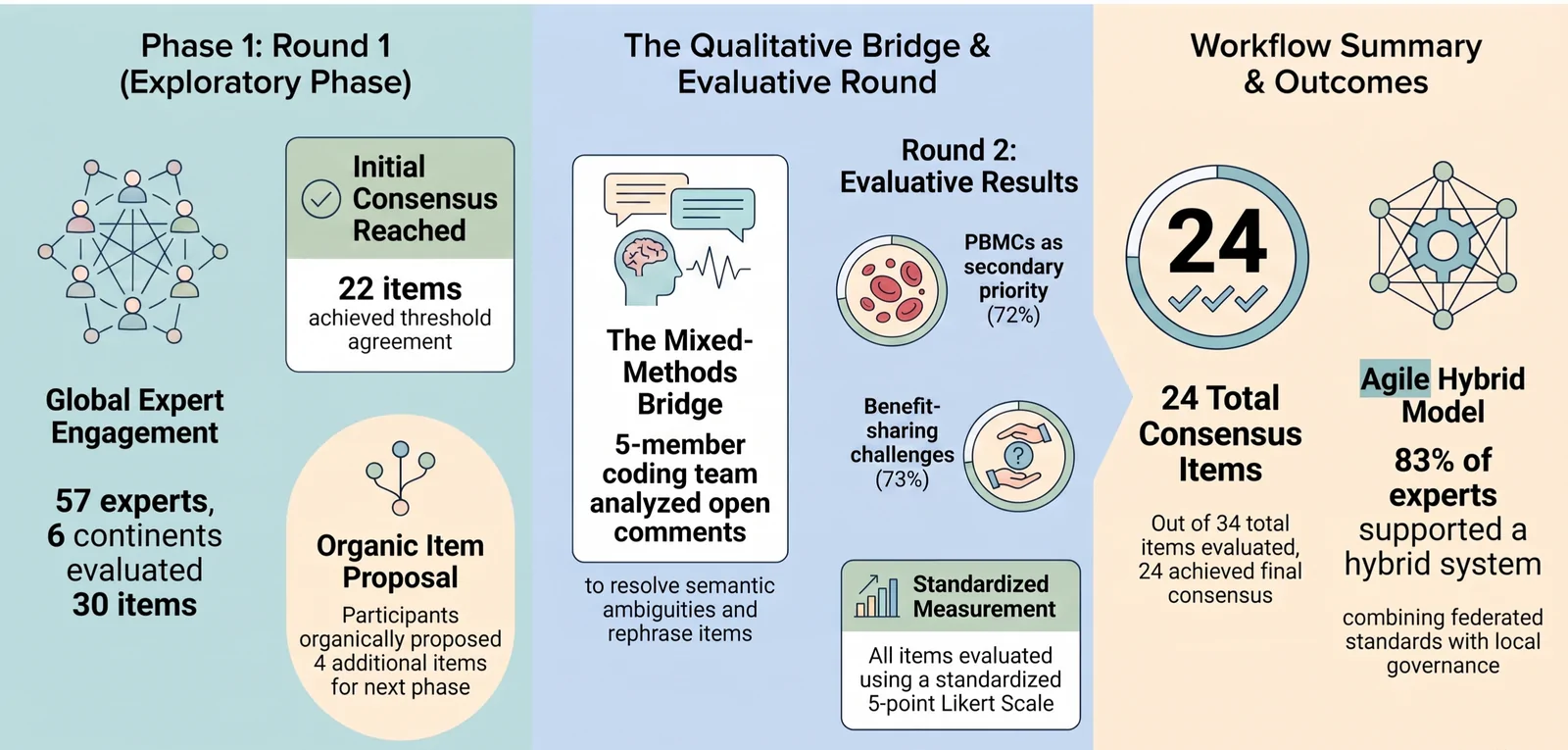
FAIR-VBS (Federated, Accessible, Inclusive, and Responsive Virtual Biorepository) mapping global consensus for virtual biorepositories (credit to Gemini and Google Notebook). PBMC: peripheral blood mononuclear cell.

We analyzed and summarized comments for both rounds in our qualitative analysis. For both rounds, we presented respondents with the quantitative responses and the comments from respondents. The items below are those that reached consensus by percentage. All detailed results including all deidentified, comments numbers or percentages, and items that did not reach consensus, are available in Multimedia Appendix 1.

### Participant Flow and Item Completeness

Panel participation followed a fluid, noninterfering sequential format. A total of 57 experts were enrolled in the initial snowball cohort. While overall survey round retention was tracked via secure login tokens, individual item denominators naturally varied across the 2 tables (ranging from n=15 to n=47). Because participants completed the panels autonomously, they frequently skipped highly technical or localized subquestions where they felt they lacked specific institutional or geographic expertise. Rather than introducing arbitrary data noise, this organic respondent flow ensures that the final consensus statistics strictly reflect the uncoerced input of qualified domain experts. Pairwise deletion was used to handle missing variables, maintaining data precision for each discrete question (Tables 1 and 2).

### Descriptive Analysis

#### General Interest and Biorepository Models

The FAIR-VBS initiative generated a high level of interest among stakeholders, with 89% (42/47) of respondents expressing either “very interested” or “extremely interested” in accessing biorepository specimens. Additionally, 85% (40/47) demonstrated strong enthusiasm for involvement in biorepository operations. When asked about preferred governance models, the combined model that integrates federated standards with local governance was favored, receiving support from 78% (37/47) of respondents. These results underscore the importance of balancing standardization with the flexibility required for diverse operational contexts.

#### Specimen Prioritization and Metadata

Blood specimens (whole blood, plasma, and serum) were overwhelmingly prioritized for collection and sharing, with 95% (40/42) of respondents ranking them as a high or very high need. The question wording related to prioritized peripheral blood mononuclear cells was clarified for the round, during which it was supported by 72% (13/18) of the respondents (Table 2). Longitudinal collections were emphasized, with 91% (40/44) supporting serial samples from confirmed cases and 84% (37/44) prioritizing prevaccination and postvaccination specimens. Additionally, 82% (36/44) highlighted the importance of sourcing specimens from endemic regions to ensure geographical diversity. Metadata accompanying specimens was deemed critical. All respondents (41/41, 100%) prioritized specimen descriptions (eg, source and type), while 91% (41/45) emphasized collection and handling conditions. Storage parameters, such as temperature and freeze-thaw cycles, were highlighted by 84% (38/45).

#### Governance and Specimen Sharing

A ranking system for specimens, based on quality and metadata, was supported by 98% (41/42) of respondents. Key ranking criteria included accessibility (36/42, 86%), completeness of clinical data (33/42, 79%), and storage and handling conditions (32/42, 76%). Barriers to specimen sharing were significant, with 77% (33/43) citing international shipping challenges.

#### Sample Quality and Metadata

Standardized quality metrics were broadly supported, with 93% (41/44) of respondents emphasizing the need for minimum quality standards. Metadata priorities included specimen descriptions (41/41, 100%) and handling conditions (41/45, 91%), reflecting the importance of robust data to support research integrity.

#### Sustainability and Services

Sustainability emerged as a pivotal concern, with unanimous support (43/43, 100%) for a cost-recovery model. A sliding fee scale, adjusted based on the type of organization (eg, academic institutions, commercial enterprises, or low- and middle-income countries), was supported by 95% (40/42) of respondents. Regarding services, 79% (34/43) prioritized reference panels for calibration and proficiency testing, while 77% (33/43) emphasized the need for protocols and templates for specimen collection and storage. Infrastructure support, such as cold chain equipment and shipping logistics, was highlighted as essential.

#### Consent and Ethics

Ethical concerns were evident in the Delphi rounds, with only 35% (13/37) affirming that broad consent, as defined by the Common Rule in 2018 [18], could be adopted within their institutions or nations for future specimen use. However, 92% (35/38) supported sharing specimens for immune-based diagnostics or vaccine development. Additionally, 84% (33/39) expressed interest in participating in policy development, reflecting a strong desire for transparency and collaboration in governance.

#### Institutional and Personal Benefits

Individual and institutional benefits did not reach consensus in round 1 or round 2. It is important to note that although 73% (11/15) acknowledged challenges with specimen-sharing benefits in round 2, no single barrier reached consensus. A lack of consensus does not necessarily indicate opposition to a particular concept. It may instead suggest that the concept is new, or poorly understood, or that respondents come from varied disciplines and cultures [19]. These variances show areas where difficult conversations may be needed with clarifications and understanding to build a FAIR-VBS that effectively meets the needs of our stakeholders [20].

#### Round 2: Refining Priorities

In round 2, full consensus was successfully achieved for 2 rephrased items that met the prespecified protocol criteria:

1. Specimen prioritization: Following panel feedback from the initial phase, respondents explicitly prioritized peripheral blood mononuclear cells as the secondary tier for biorepository accrual, reaching 72% consensus (13/18).
2. Barriers and benefit-sharing: Respondents strongly confirmed the systemic friction points surrounding resource equity, with 73% (11/15) reaching consensus that clear institutional challenges exist to effectively receive specimen-sharing benefits.

Consensus was not reached concerning the responsibility of the VBS for preserving SARS-CoV-2 specific pathogens. Detailed tracking of all items that failed to reach consensus is archived transparently within Multimedia Appendix 1.

### Delphi Participant Comment Inclusion

To ensure thematic integrity, a collaborative, multicoder qualitative content analysis was integrated into the iterative Delphi rounds. Textual data from the open-comment fields were evaluated independently by a core coding team of 5 researchers (AP, LA, MC, JG, and JP). The team used a consensus-driven coding framework centered on our 3 target domains: specimen prioritization, structural barriers, and resource sustainability. All researchers had current ethics training and practice and received further instruction on Delphi method coding in compliance with ACCORD guidelines.

To prevent steering group bias or top-down paternalism, discrepancies in theme categorization were resolved through a formal reconciliation process where a team agreement threshold of 100% was required before a code was finalized. This qualitative engine served a strict procedural purpose between rounds: when an item failed to achieve quantitative consensus in round 1, its associated open-text codes were systematically used to uncover semantic ambiguities. The steering group then used these exact participant insights to structurally rephrase the items for clear evaluation in round 2, guaranteeing that the subsequent consensus rounds strictly reflected uncoerced, organic stakeholder intent. All comments were deidentified and shared with all participants in the form in which they were written to reduce unintentional bias. Please note that these comments may contain perceptions that are not representative of consensus. This is common in emergent fields and is helpful in finding unintentional pain points, biases, and errors.

### Open Comments Summary

#### Specimen Prioritization

Respondents reinforced the prioritization of blood specimens (whole blood, plasma, and serum) for collection, storage, and sharing. Other suggestions included cerebrospinal fluid, tissue, and breast milk. Feedback emphasized that specimen prioritization should adapt to specific pathogens, with strong support for longitudinal sampling.

#### Barriers to Specimen Sharing

Shipping challenges were the most frequently cited obstacle (11/15, 73%), compounded by infrastructure limitations, especially in regions with inadequate cold-chain capacity. Regulatory complexities, such as the Nagoya Protocol [21], were also noted as a barrier to international sample sharing.

#### Governance: Federated Standards With Local Adaptation

The combined governance model, integrating federated standards with local oversight, emerged as the most effective approach. Respondents highlighted the advantages of local governance in enabling rapid responses, particularly during pandemics, while federated standards ensure consistency across regions.

#### Ethical and Policy Considerations

Local and national regulations posed obstacles, as identified by multiple respondents. This highlights the need for streamlined logistics and regulatory frameworks to facilitate international collaboration.

#### Sustainability and Recommendations

Funding was identified as a major challenge, with respondents stressing the need for long-term coordination and infrastructure development. Recommendations included establishing training programs to build equity in lower-resourced settings and incorporating standard operating procedures. Notably, participants suggested engaging more stakeholders from regions that are underrepresented in the current survey, particularly Africa.

The Delphi survey results emphasize the importance of a flexible yet standardized approach to biorepository governance, robust quality standards, and inclusive stakeholder collaboration. These findings will guide the next stages of developing the FAIR-VBS, ensuring it is equitable, sustainable, and responsive to global research needs (Figure 4).

**Figure 4.**
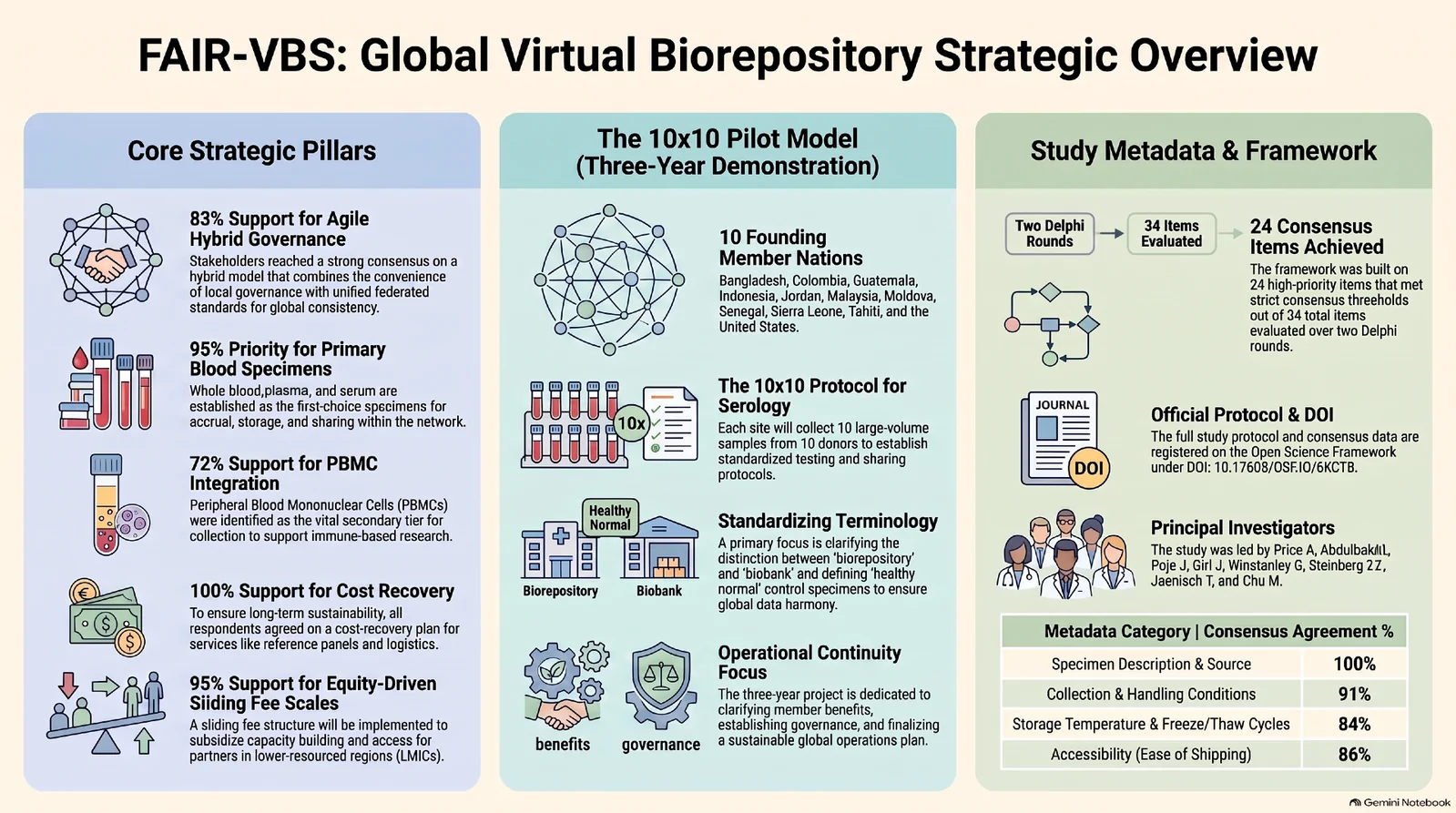
FAIR-VBS (Federated, Accessible, Inclusive, and Responsive Virtual Biorepository) building consensus and priority needs (credit to Gemini and Google Notebook).

## Discussion

### Principal Findings

The Delphi method revealed a rich tapestry of insights about the potential of a FAIR-VBS. The enthusiasm among respondents not only highlighted the transformative possibilities but also brought to light critical areas that must be addressed to ensure its success. One of the most striking benefits of the FAIR-VBS lies in its ability to foster international collaboration. Respondents expressed optimism about how the system could break down traditional barriers in sample sharing, facilitate meaningful cross-border partnerships, and provide opportunities for training and capacity building. This is particularly exciting for underresourced regions, where the FAIR-VBS could play a pivotal role in reducing disparities and promoting equity in global health research [1].

A key strength of the system lies in its ability to connect users with biorepository expertise. By aligning operations with international standards and developing standard operating procedures with quality requirements [22], the FAIR-VBS can elevate the reliability of biospecimen management for infectious disease research worldwide. Respondents envisioned a future with the FAIR-VBS as a cornerstone for consistent, high-quality international research.

Nevertheless, this vision comes with its challenges. A notable emergent theme was the regulatory knowledge gap across countries, highlighting the need for codeveloped resources to align understanding of local and national regulations. This is particularly relevant to the application of benefits under the Nagoya Protocol, as outlined in the Convention on Biological Diversity, which specifies explicit requirements for signatory countries [21]. While this lack of shared knowledge could hinder cross-border collaborations, it also offers an opportunity to develop a system that addresses these gaps through accessible guidance and coproduced documentation.

Concerns about infrastructure and resource limitations were prominent in the discussions. Stable power supplies, reliable storage solutions, and contingency plans for system failures are essential for maintaining sample integrity [22]. Sustainable funding models were another area of concern, as respondents noted the challenges of securing long-term financial support for infrastructure, supplies, and personnel. These issues underline the importance of a robust operational foundation to support the FAIR-VBS’s goals.

Logistics posed another layer of complexity, particularly considering past experiences during outbreaks. Shipping delays, high costs, and cumbersome regulations were frequently cited as barriers that needed to be addressed. Respondents called for innovative solutions to streamline these processes, ensuring that the movement of specimens is both timely and cost-effective.

The discussions also explored the unique difficulties of operating in conflict zones or disaster-stricken areas. Strategies to improve access in such environments, such as establishing proxy centers, were proposed as potential solutions to ensure inclusivity and equity.

Ethical considerations emerged as a critical theme, with respondents advocating for careful negotiation of broad consent frameworks [23]. The balance between enabling research and respecting participants’ rights was a shared priority, with consensus around the need to limit consent to intended uses and avoid misuse by third parties [23]. Another area of concern was the power dynamics inherent in global collaborations. Smaller biorepositories often face challenges in negotiating on equal terms with larger entities, highlighting the need for governance structures that prioritize fairness and inclusion. Similarly, the importance of equitable benefit-sharing was universally acknowledged, though the variability in challenges across regions pointed to the complexity of designing solutions that work for everyone.

Finally, questions about data and sample quality underscored the importance of consistency and rigor. Ensuring accurate clinical data, maintaining high diagnostic standards, managing samples effectively, and providing timely reference materials were seen as vital to the FAIR-VBS’s success [24]. These discussions point to a powerful vision for the FAIR-VBS, one that could transform global health research by promoting collaboration, equity, and innovation. Innovations in safe and secure storage materials that are free of expensive ultralow cold storage could alleviate costs for power, equipment, and shipping. Yet, the path forward requires thoughtful, inclusive strategies to address the challenges and ensure the system’s benefits are accessible to all. By fostering transparency, sustainability, and shared ownership, the FAIR-VBS has the potential to become a model for how global health infrastructure can evolve to meet the needs of an interconnected world.

### Challenges and Limitations

The first Delphi round found broad support for a combined biorepository model, incorporating federated standards with local governance to optimize standardization and agility. This approach can address administrative delays inherent in federated systems while allowing for the rapid mobilization of resources necessary for public health emergencies. Consensus was reached on specimen types and quality standards, although defining the normal sample, along with education about why this is critical for research and practice, will be a priority for the FAIR-VBS. Normal control or reference samples may bring the fewest legislative hurdles and could be an appropriate foundation for testing intercountry sharing of specimens, as they are not subject to the same restrictions as infected samples. These samples can also serve as a testing ground for defining terms and differences. Although 1 participant expressed concerns that people living in Africa and working in laboratories were expatriates rather than native, locally educated African individuals, we acted on this assumption by ensuring that native, locally educated people would lead or colead the repositories, thus ensuring local country representation and protection for the laboratories should evacuation or government unrest, or conflict occur. One of the goals of the FAIR-VBS is to ensure strong, trained, and local representation in each country.

The Delphi process allowed us to further query our stakeholders concerning institutional and personal benefits. We found that differing national policies, localized terminology barriers, and ambiguities regarding exactly who would benefit (eg, local collection sites, national governments, or the centralized FAIR-VBS infrastructure) were prominent friction points. Rather than forcing an artificial consensus by shifting our predetermined analytical standards, which would compromise scientific precision, we used our iterative multicoder qualitative framework to map these underlying anxieties.

Recognizing these structural barriers, our ongoing implementation project explicitly codeveloped localized solutions. Specifically, FAIR-VBS sites requested a low operational entry bar featuring an equitable, sliding fee scale tailored for underresourced centers unable to absorb additional costs. Furthermore, stakeholders required a binding assurance that all protocol features remain entirely compatible with their unique national regulatory standards and be mutually agreed upon by all global partners. This participant-driven approach ensures complete data sovereignty while building international trust in emergent biobanking architectures.

While snowball sampling was highly practical for rapidly engaging a specialized global network, it can introduce inherent network bias. This methodology may limit the overall generalizability of the final consensus due to the potential overrepresentation of specific professional networks, which inherently shapes the demographic and professional diversity of respondents. Language barriers were discussed. We learned that other language respondents were comfortable in filling out the Delphi in English; however, respondents shared that language barriers are more challenging to overcome in oral discussions. Ongoing discussions are needed to address these logistical and ethical challenges. Benefits sharing will require investigation and collaboration locally and within the federated system. Round 2 highlighted the need for reaching people native to the countries, rather than only expatriates assigned to the regions. Respondents expressed anticipated hesitation concerning the FAIR-VBS role and structure, and these findings will guide future discussions as we continue to refine the FAIR-VBS model. As this is an exploratory and participatory Delphi study, we do not pretend to be representative of biorepositories at large and generalizability is limited. For the same reasons, we are not equipped to discuss international rulings or the benefits or barriers of the Nagoya Protocol. Our purpose in asking these questions was to query how this protocol impacted this international sample. We are unpacking nonconsensus items; however, this information is not yet ripe for publication.

### Future Directions

Guided by the Delphi findings, we have launched a 3-year demonstration project involving representatives from 10 countries (Bangladesh, Colombia, Guatemala, Indonesia, Jordan, Malaysia, Moldova, Senegal, Sierra Leone, and Tahiti) and the United States (as the founding and currently facilitating country). This initiative, built around the “10 × 10” model—10 large-volume samples from 10 donors per site for serology testing, focuses on governance, member benefits, and a sustainable operations plan. Early efforts prioritize standardizing terminology, such as clarifying “biorepository” vs “biobank,“ [25,26] and defining “healthy normal” to ensure consistency across sites.

Reflecting respondents’ preferences, the project emphasizes ethical sharing, sustainable access, and locally guided sample collection, supported by a buddy system to expand membership. Contingency plans include sharing calibrated test kits and harmonized standards across sites when specimen sharing proves impractical. During the period between manuscript review and acceptance, our team acted on the Delphi findings. These coordinated efforts aim to advance the FAIR-VBS as a global platform for equitable, ethical, and collaborative specimen sharing.

### Conclusions

These findings highlight the transformative potential of the FAIR-VBS to address critical gaps in international biorepository efforts and the significant challenges that remain to be navigated for implementation. The enthusiasm for enhanced collaboration and capacity building aligns with the FAIR-VBS’s mission to promote inclusion and equity in specimen sharing. At the same time, the complexities of infrastructure, regulations, and logistics underscore the need for a strategic and comprehensive approach. Concerns about power dynamics and data quality call for robust and respectful cocreated governance frameworks and agreed-upon quality-control measures. Future efforts will focus on practical solutions, including regulatory harmonization, infrastructure development in resource-limited settings, and equitable governance structures. Pilot work will provide actionable insights into overcoming these challenges. The FAIR-VBS global equity slideshow is presented in Multimedia Appendix 1.

## Supplementary material

10.2196/87458 Multimedia Appendix 1 Federated, Accessible, Inclusive, and Responsive Virtual Biorepository global equity blueprint.

## Abbreviations

ACCORD: Accurate Consensus Reporting Document
CHERRIES: Checklist for Reporting Results of Internet E-Surveys
CUREC: Central University Research Ethics Committee
FAIR-VBS: Federated, Accessible, Inclusive, and Responsive Virtual Biorepository
ICMJE: International Committee of Medical Journal Editors
